# Early onset nephrotic syndrome with dysmorphic facies and microcephaly

**DOI:** 10.12860/jnp.2015.19

**Published:** 2015-07-01

**Authors:** Muhammed Mubarak, Ali Lanewala, Seema Hashmi

**Affiliations:** ^1^Histopathology Department, Sindh Institute of Urology and Transplantation, Karachi, Pakistan; ^2^Nephrology Department, Sindh Institute of Urology and Transplantation, Karachi, Pakistan; ^3^Department of Pediatric Nephrology, Sindh Institute of Urology and Transplantation, Karachi, Pakistan

**Keywords:** Infantile nephrotic syndrome, Galloway-Mowat syndrome, diffuse mesangial sclerosis

**Implication for health policy/practice/research/medical education:**

Early-onset or infantile nephrotic syndrome (NS) may be associated with a number of systemic syndromes. It is imperative to diagnose the underlying syndrome correctly as it has considerable therapeutic implications. Given the rarity of such syndromes, it is mandatory to document fine phenotypic details of such cases to increase the understanding of such disorders.

## Case Presentation

A 14-month-old girl, presented with history of generalized body swelling at the age of 9 months, which progressed to anasarca. No specific treatment for the ailment was given. She is the second child of a consanguineous couple, the first child also being 3 years old girl and healthy. Family history was negative for renal diseases. Mother denied any illness during her pregnancy and her natal and immediate postnatal period was unremarkable. An ultrasound done at seventh months of gestation was unremarkable. Her perinatal and neonatal periods were unremarkable.

She was developmentally normal before the onset of edema and was able to sit without support at 9 months of age, with social smile and recognition of parents and other family members; however, over the next 5 months with worsening edema, she regressed and at the time of presentation, she was very irritable, had lost her social smile and could not even hold her neck.

There was no history of preceding febrile illness, rash, hematuria or seizures and she had received her scheduled immunizations with bacillus Calmette–Guérin (BCG), hepatitis B, polio, diphtheria, tetanus, pertussis (DTP) and hemophilus influenzae type b (Hib).

**Figure 1:**
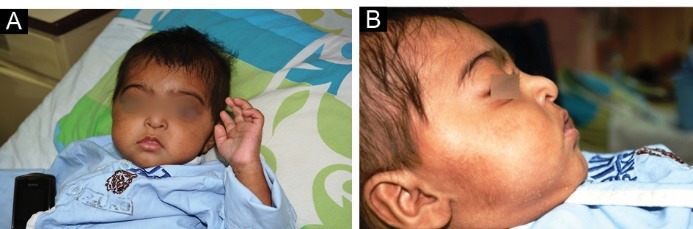
A) Facial features of the child, front view. B) Lateral view of the face.

On general physical examination, her length (64 cm) and head circumference (38 cm) were below third percentile for age while her weight (with gross anasarca) (5.7 kg) was between 5th to 10th percentiles. Her facial features are shown in Figure 1A and 1B. Her blood pressure was 67/43 mm Hg. The ophthalmologic examination by a pediatric ophthalmologist showed mega eyeballs, pin-point pupils, severe myopia and loss of foveal reflex. The abdominal examination showed gross ascites and the fingers, arachnodactyly. Examination of the nervous system revealed no sensory or motor defects. No focal deficit was elicited. Cranial nerves were intact. Rest of her physical examination was unremarkable.

Ultrasound abdomen showed moderate ascites and no hepatosplenomegaly. Both kidneys were normal in size.

Her baseline investigations showed serum urea, 9 mg/dl; serum creatinine, 0.46 mg/dl; low serum albumin (1.2 g/dl) with spot urinary protein to creatinine ratio of 6.1; serum cholesterol, 143 mg/dl; serum total proteins, 3.4 g/dl; C3, 0.5 g/l and C4, 0.12 g/l. Microscopic hematuria (3+) was detected but there was no glycosuria.

**Figure 2:**
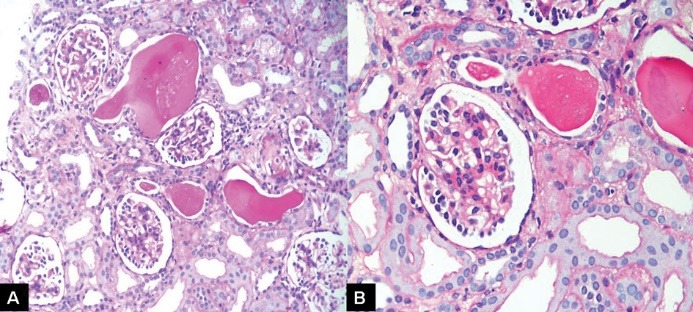
Representative sections of the renal biopsy.

**Figure 3:**
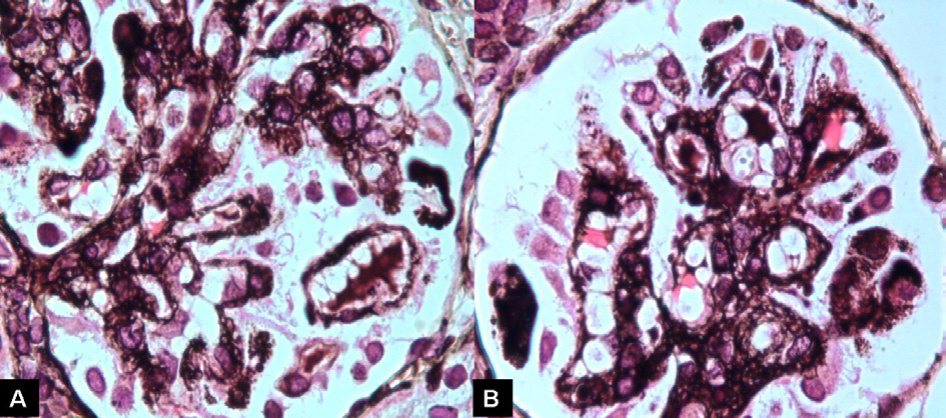
Glomerular lesions on high-power view.

She was administered 2 mg/kg/day of prednisolone for 4 weeks with no significant reduction in proteinuria. She was also given intravenous furosemide as diuretic agent which resolved her anasarca. At 4 weeks after first presentation, she was clinically diagnosed as steroid-resistant nephrotic syndrome (SRNS) and the steroid dose was decreased to 1 mg/kg/day every other day and the renal biopsy was performed. The representative pictures of the renal biopsy are shown in Figures 2 and 3. She was started on angiotensin-converting enzyme inhibitor (ACEi), 2.5 mg, once a day and started to show improvement in urinary protein spillage.

**Figure 4:**
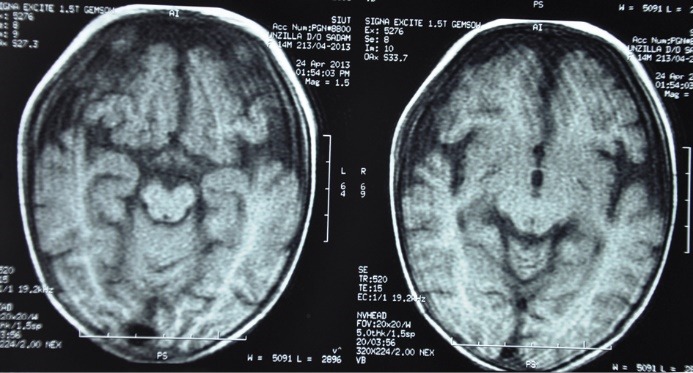
Representative MRI images of the child.

Her skeletal survey was normal. The magnetic resonance imaging (MRI) of brain was done and is shown in Figure 4. Genetic testing revealed no mutations in *NPHS1* and *NPHS2* genes. Her cytogenetic analysis could not be done due to non-availability of the test.

Incidentally, within one week of renal biopsy, she developed left thigh abscess, which ruptured spontaneously and large amount of pus was drained. She was empirically started on ceftriaxone, 300 mg once a day. Culture grew *pseudomonas* and the antibiotic was changed to imipenem according to the sensitivity pattern and she responded dramatically. Interestingly, following the resolution of the abscess, her edema disappeared completely and proteinuria decreased to 2+ on dipstick testing (partial response). Her steroids were tapered and gradually withdrawn. She was discharged on ACEi. Her renal functions remained normal throughout and the last serum creatinine was 0.34 mg/dl, which is 8 months postrenal biopsy (23 months of age). Urine dipstick examination revealed 1+ protein, and no hematuria. She was edema free at the last follow-up.

## Questions

1. Describe the differential diagnosis of the condition.
2. Describe the facial features of the patient.
3. Describe the renal biopsy findings.
4. Describe the MRI findings in this case.
5. What is the Galloway-Mowat syndrome?

## Answers

1. This is the case of an early or infantile-onset nephrotic syndrome (NS). Infantile NS, like congenital NS (CNS) may be primary or secondary to a variety of infectious diseases (eg, syphilis, toxoplasmosis, etc). Primary infantile NS, like CNS, is mostly caused by mutations in genes coding for podocyte proteins. These cases are usually steroid resistant. In this child, the NS is associated with microcephaly and facial dysmorphism, as well as developmental retardation. However, the later was of late-onset and nonprogressive. The concurrence of NS with microcephaly and developmental delay may be coincidental, or may represent one of at least three syndromes: Galloway-Mowat syndrome (GMS), a second syndrome of microcephaly, NS and developmental delay (MNSDD), and a third syndrome of microcephaly, developmental delay, and spondylorhizomelic short stature.
2. A prominent facial dysmorphism is seen in some hereditary forms of the NS. A careful study of the facial features can provide useful clues for the accurate diagnosis of the underlying renal pathology. The head and neck examination from a front view of this child shows microcephaly, coarse hair, narrow forehead, large, hypertelorism, and almond-shaped eyes. There is also arachnodactyly (Figure 1A). The lateral view of the face shows large, low-set ears, depressed nasal bridge, thin lips, and micrognathia (Figure 1B).
3. The percutaneous renal biopsy comprised of single core of renal cortex. Up to 50 glomeruli were included. The representative sections showing glomerular and tubular changes are shown in Figure 2. Of all, 15 were primitive, rest showed diffuse mesangial proliferation, chiefly of the matrix, and focally, the cells (Figure 2A and 2B). Some glomeruli also show focal inter-positioning into peripheral capillary loops, producing tram-track appearance. The expanded mesangial areas show reticulated fibrosis and sclerosis (Figures 3A and 3B). Podocytes are prominent in many glomeruli producing crown-like formation. Glomerular basement membrane (GBM) is thickened and fuzzy in outline and reduplicated in some capillary segments. Mild patchy tubular atrophy and interstitial inflammation are seen. Microcystic dilation of a few tubules with hyaline cysts is also noted (Figure 1A). Immunofluorescence was completely negative for IgG, IgA, IgM, C3 and C1q. The immunomorphological features on the renal biopsy are consistent with diffuse mesangial sclerosis (DMS).
4. MRI of the brain shows generalized dilatation of the extra axial cerebrospinal fluid (CSF) spaces, which represents diffuse cortical atrophy (DCA). Brain parenchyma including cortex, basal ganglia and white matter are unremarkable.
5. Galloway and Mowat in 1968 described 2 sibs with early onset NS, microcephaly, and hiatus hernia (1). This combination of findings received the nomenclature of GMS. Since 1968 at least 50 cases of the syndrome have described with ever expanding spectrum of phenotypic findings. However, genotypic abnormalities underlying the syndrome are still elusive. It is not entirely clear whether the expanding phenotypic abnormalities represent heterogeneities within the syndrome or simply the different stages in the evolution of the disease (2-5). There is also some phenotypic overlap with another well-known syndrome of early-onset NS and ocular anomalies, the Pierson syndrome (PS). This has resulted in some confusion about the nature and distinctiveness of GMS, further aggravated by the lack of knowledge of the genes or molecules involved in this syndrome. GMS is however, believed to be transmitted in an autosomal recessive pattern. Some authors believe it to be primarily a GBM disorder with marked ultrastructural changes of GBM on electron microscopy (EM).

## Authors’ contribution

All authors contributed equally to the preparation of this manuscript.

## Conflicts of Interest

The authors declared no competing interests.

## Funding/Support

None.
